# Comparison of Different LGM-Based Methods with MAR and MNAR Dropout Data

**DOI:** 10.3389/fpsyg.2017.00722

**Published:** 2017-05-12

**Authors:** Meijuan Li, Nan Chen, Yang Cui, Hongyun Liu

**Affiliations:** ^1^Collaborative Innovation Center of Assessment Toward Basic Education Quality, Beijing Normal UniversityBeijing, China; ^2^Educational Supervision and Quality Assessment Research Center, Beijing Academy of Educational SciencesBeijing, China; ^3^School of Psychology, Beijing Normal UniversityBeijing, China; ^4^Beijing Key Laboratory of Applied Experimental Psychology, School of Psychology, Beijing Normal UniversityBeijing, China

**Keywords:** latent growth model, missing at random (MAR), missing not at random (MNAR), Diggle–Kenward selection model, maximum likelihood approach

## Abstract

The missing not at random (MNAR) mechanism may bias parameter estimates and even distort study results. This study compared the maximum likelihood (ML) selection model based on missing at random (MAR) mechanism and the Diggle–Kenward selection model based on MNAR mechanism for handling missing data through a Monte Carlo simulation study. Four factors were considered, including the missingness mechanism, the dropout rate, the distribution shape (i.e., skewness and kurtosis), and the sample size. The results indicated that: (1) Under the MAR mechanism, the Diggle–Kenward selection model yielded similar estimation results with the ML approach; Under the MNAR mechanism, the results of ML approach were underestimated, especially for the intercept mean and intercept slope (μ_*i*_ and μ_*s*_). (2) Under the MAR mechanism, the 95% CP of the Diggle–Kenward selection model was lower than that of the ML method; Under the MNAR mechanism, the 95% CP for the two methods were both under the desired level of 95%, but the Diggle–Kenward selection model yielded much higher coverage probabilities than the ML method. (3) The Diggle–Kenward selection model was easier to be influenced by the non-normal degree of target variable's distribution than the ML approach. The level of dropout rate was the major factor affecting the parameter estimation precision, and the dropout rate-induced difference of two methods can be ignored only when the dropout rate falls below 10%.

## Introduction

A longitudinal study involves repeated observations of properties of an individual over a period of time to explore the characteristics of the emergence, development, and change of the properties. In contrast to cross-sectional analysis, a longitudinal study contains information on the properties that vary with time, and thus can examine the change process of a particular property over time and make more reasonable inferences of the causal relationships between different variables. With the development of statistical techniques and as studies become more complex, the longitudinal data analysis method has drawn increasing attention. In longitudinal studies, despite the efforts of researchers to maintain the same sample throughout the process, the time-consuming nature of such studies results in a common scenario where subjects might quit the experiments because of individual properties or other external factors, resulting in large amounts of missing data. Although a common problem for researchers in longitudinal studies, the appropriate methods of handling missing data are difficult to choose. A review of 100 longitudinal studies conducted by Jelicic et al. (2009) found that 57 contained missing data and that the approaches used by 87% of these studies were inappropriate.

The choice of method to handle the missing data is dependent on the mechanisms that lead to missing data and missingness patterns. Little and Rubin (2002) came up with three missing data mechanisms: (1) missing completely at random (MCAR), the probability of missing data on a variable Y is unrelated to other observed variables in the data set, and also unrelated to the values of Y itself; (2) missing at random (MAR), the probability of missing data on a variable Y is related to other observed variables in the data set, but unrelated to the values of Y itself; (3) missing not at random (MNAR), the probability of missingness on Y depends on the values of Y itself. A common problem with analyzing longitudinal data is that subjects may have dropped out of the study prematurely in such a way that ignoring the mechanism for dropout will lead to biased estimates and standard errors. In such situations, the dropout mechanism is called “non-ignorable” (Little and Rubin, 2002). Whether missing data is considered non-ignorable depends on the method of analysis, specifically which types of missingness it can account for. In likelihood based estimation, MNAR is non-ignorable (Power et al., 2012).

Research on missing data methods has been an area of interest in recent decades. Large number of studies indicated that some of the simple methods commonly used by researchers for handling missing data, such as list-wise deletion (LD), pairwise deletion, and single imputation (for example, mean substitution, regression substitution among others), are beset with limitations, such as biased parameter estimates and reduced testing power, and are therefore not recommended (Jelicic et al., 2009; Enders, 2010). Over the past decade, studies on missing data methods have focused on discussions of the approaches under the MAR mechanism. Multiple imputation and maximum likelihood estimation (MLE) are two of the methods that are most widely used and frequently recommended (Carpenter et al., 2006). MLE is a model-based method for handling missing data, while multiple imputation is a distribution-based multiple data replacement method. Researchers have also come up with a range of methods for MNAR data in recent years (Wu and Carroll, 1988; Wu and Bailey, 1989; Diggle and Kenward, 1994; Follmann and Wu, 1995; Molenberghs and Kenward, 2007; Molenberghs et al., 2009; Enders, 2011a).

For MNAR data, owing to the need to describe the relationship between the missingness mechanism and the target variable, the methods used are mostly model-based, i.e., to make an analysis by defining the mechanism that leads to the missing data (Little and Rubin, 2002; Enders, 2010). For analysis of longitudinal data with MNAR-values, the practice is to add a model to describe the characteristics of the missing data based on the growth model (i.e., subjects response pattern or development curve) to correct the bias (Ye et al., 2014). Selection modeling was first applied by Heckman (1976) to handle longitudinal data with MNAR-values and has since attracted wide interest and attention among methodologists. Little and Rubin (2002) and Schafer and Graham (2002) recommended the use of selection modeling and pattern-mixture modeling to deal with MNAR data. A number of selection models and pattern-mixture models for handling MNAR data were derived based on the latent growth model (LGM), such as Wu–Carroll (1988) model and Diggle–Kenward (1994) selection model. The incorporation of latent class variables into these models has enabled better operations of growth models with MNAR data. Examples of these models include latent class selection model (Beunckens et al., 2008), Diggle and Kenward latent class selection model (Muthén et al., 2011), Roy (2003) latent class pattern-mixture model, Muthén-Roy latent class pattern-mixture model (Muthén et al., 2011) among others. In recent years, these model-based MNAR approaches have been increasingly used in data analyses of longitudinal studies (Enders, 2011b; Muthén et al., 2011; Power et al., 2012), especially in clinical studies. For example, Enders (2011b), Muthén et al. (2011), and Power et al. (2012) all employed LGMs to analyze the clinical trial data in mental illnesses treatments. They also conducted sensitivity analyses by using the ML method, Diggle–Kenward selection model, Wu-Carroll model (Wu and Carroll, 1988) and conventional pattern-mixture models as well as latent class selection model (Beunckens et al., 2008), Diggle–Kenward latent class selection model (Muthén et al., 2011), Roy (2003) latent class pattern-mixture model, and Muthén-Roy latent class pattern-mixture model (Muthén et al., 2011) that took the impact of latent classes into consideration.

Although a variety of applicable methods for dealing with MNAR data have been proposed and applied in actual research, researchers are still finding it difficult to make appropriate choices. First, all types of model-based approaches under the MNAR mechanism need to satisfy certain assumptions. For example, the Diggle–Kenward selection model (Diggle and Kenward, 1994) requires normal distributional assumptions for the repeated measures variables. Otherwise, the model is inestimable. With continuous outcomes, the typical practice is to assume a multivariate normal distribution for the individual intercepts and slopes or for the repeated measures variables. Some researchers noted that MNAR-based approaches might be more sensitive to the missingness mechanism and assumptions on normal distribution (Enders, 2011a,b). However, no evidence supports this conclusion. Further study is still needed to decide the robustness of these model-based approaches under the MNAR mechanism when their assumptions cannot be satisfied (i.e., when data are non-normal).

In addition, controversy exists over the selection of approaches under MNAR mechanism in reality. Some researchers argued that to ignore the MNAR mechanism and use MAR-based methods instead could lead to biased estimates. However, others believed that a good MAR model, even with violated assumptions, would still be better than an ill-designed MNAR model (Schafer, 2003). Tacksoo et al. (2009) conducted a comparison of the methods for handling non-normal data with MNAR-values to confirm that the LGM-based robust MLE method yielded strikingly better results than the LD and the pairwise asymptotically distribution-free method (pairwise ADF). Nevertheless, the methods compared in the study all required that data should be MAR, and there was no discussion on whether the robust MLE method was robust in dealing with MNAR data. Therefore, if the MAR-based robust MLE method is adopted under the MNAR mechanism, can it perform as good as the MNAR-based modeling methods? Furthermore, if MNAR-based modeling methods are adopted under the MAR mechanism, will the results be different from MAR-based robust MLE? As there are currently no clear data available to test the MAR and MNAR mechanisms, a discussion on these questions will be of great value to the selection of missing data methods in practical longitudinal studies.

Based on the above questions, this study focus on the following questions using Monte Carlo simulation study: Under different missingness mechanisms, will the growth model-based ML method and the Diggle–Kenward selection model yield different results? When dealing with these questions, the study takes into account the influences of the skewness and kurtosis of data, the dropout rate and the sample size on the different methods, and provides recommendations accordingly for the selection and use of methods.

## Growth model-based methods for dealing with missing data

**Figure 1:**
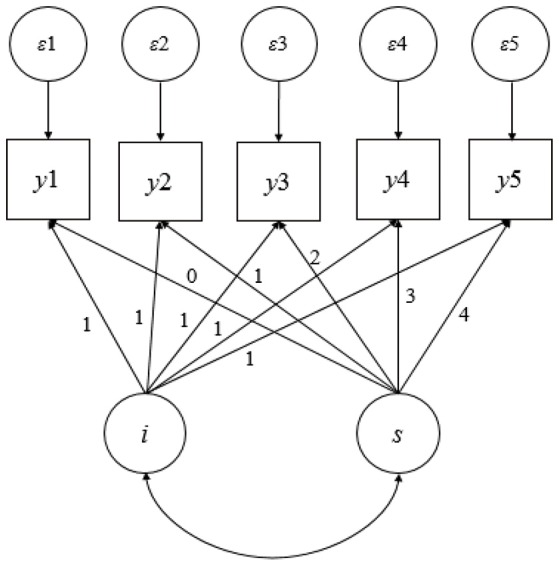
**Illustration of a latent growth model**.

This study was based on LGM for longitudinal data analysis, and a brief overview of the LGM is warranted before proceeding. Figure 1 shows a path diagram of a linear growth model from a longitudinal study with five equally spaced assessments. The unit factor loadings for the intercept latent variable reflect the fact that the intercept is a constant component of each individual's idealized growth trajectory, and the loadings for the linear latent variable capture the timing of the assessments. A number of resources are available to readers who want additional details on LGMs (Singer and Willett, 2003; Bollen and Curran, 2006).

### MAR-based maximum likelihood method

Based on the LGM model, the ML method under the MAR assumptions is a model-based approach for dealing with missing data, which defines a model for the observed data and makes inferences based on the likelihood function. The ML method does not require imputation of unobserved data during the model fitting process; instead, it uses the information of the observed variable values to conduct parameter estimations as a way of handling missing data. When the ML method is used to deal with missing data, the joint distribution of the target variable *Y*, and the missingness indicator *d*, *f* (*Y*_*i*_, *d*_*i*_|θ, ϕ), where *Y*_*i*_ is the target observations of the *i*th individual, *d*_*i*_ is an indicator variable used to describe whether the observation of the *i*th individual is missing, θ is the parameter of latent growth model, and φ is a parameter used to describe missingness mechanism. The function *f* is simply factorized into the product of two independent distributions without the need to estimate the parameter (φ) for predicting the probability of missingness.

As the ML method can yield unbiased parameter estimates under the MAR mechanism and is more efficient than other conventional methods (e.g., LD, pairwise deletion, single imputation), methodologists have considered it a “state-of-art missing data technique” (Schafer and Graham, 2002). Even under the MCAR mechanism, the ML method can still yield better statistics than other methods since it can achieve maximized estimation efficiency by “borrowing” information from observed data (Enders and Bandalos, 2001). Although the ideal MLE assumes that data are normally distributed, a large number of studies show that the robust MLE with a non-normality correction (Yuan and Bentler, 2000) can also produce approximately unbiased results even under non-normality cases. However, the performance of this approach under the MNAR assumptions is not yet clearly elucidated.

## Methodology

### Simulation design

To examine the factors that influence model parameter estimations, this study consulted the factors considered in the simulation studies conducted by former researchers (Newman, 2003; Kristman et al., 2005; Gad and Ahmed, 2007; Mazumdar et al., 2007; Langkamp et al., 2010; Soullier et al., 2010; Yuan et al., 2012) and came up with a simulation design as follows:

1. Sample size. In light of the recommendations of previous studies (Zhang and Willson, 2006) that growth models should have a minimum sample size of 50, the present study chose four sample levels at 100, 300, 500, and 1,000, respectively.
2. Dropout rate. Four drop rate levels were chosen, i.e., 5, 10, 20, and 40%.
3. The degree of non-normal distribution of the target variable. Four levels of skewness and kurtosis were considered. In light of the degrees of non-normal distribution adopted by previous simulation studies (Yuan and Bentler, 2000; Enders, 2001; Tacksoo et al., 2009; Yuan et al., 2012), the skewness and kurtosis were set at 0 and 3 for normal data, 0.5 and 6 for the slightly non-normal case, 2 and 15 for the moderately non-normal case, and 3 and 33 for the extremely non-normal case, respectively.
4. Two missingness mechanisms, i.e., MNAR and MAR, were involved.

The study included a total of 4 × 4 × 4 × 2 = 128 simulation conditions. Analysis was repeated on each condition for 500 times using the MAR-based robust MLE method and the MNAR-based Diggle–Kenward selection model.

### Data generation

The study consulted the data generation methods for different types of missing data used by Tacksoo et al. (2009) and employed the R language to generate longitudinal data sets that satisfied different model assumptions and contained different missingness mechanisms. The process of data generation is as follows:

First, the study generated a complete set of longitudinal data. The simulated data set represented a longitudinal study on a number of *n* subjects where measurement was repeated for *t* (*t* = 5) times, yielding an observed value each time. An LGM was used to generate the observed value *y*_*j*_ of each subject at each time point that satisfied the distribution features of the target variable, where *j* = 1, …, *t*. The parameters of the LGM were set as follows:

$$\begin{array}{l} \text{the intercept},\text{ i}\sim \text{N}\left(-1,0.50\right)\\ \text{the linear slope},\text{ s}\sim \text{N}\left(0.5,0.02\right)\text{ and}\\ \text{the residuals},\;\left(\begin{array}{c} \varepsilon_{1}\\ \varepsilon_{2}\\ \varepsilon_{3}\\ \varepsilon_{4}\\ \varepsilon_{5} \end{array}\right)\sim \text{N}\left(\begin{array}{c} 0.50\\ 0.48\\ 0.42\\ 0.32\\ 0.18 \end{array}\right) \end{array}$$

Non-normal distribution data with specific skewness and kurtosis were generated using the generalized Lambda distribution (GLD) (Headrick and Mugdadi, 2006). The GLD family is defined by the following inverse distribution function:

$$\begin{array}{l} F^{-1}\left(y\right)=\lambda_{1}+\frac{y^{\lambda_{3}}-{\left(1-y\right)}^{\lambda_{4}}}{\lambda_{2}} \end{array}$$

where 0 ≤ *y* ≤ 1. The mean, variance, skewness, and kurtosis of the distribution can be expressed as the following formulas:

$$\begin{array}{l} \alpha_{1}=\mu \;=\;\lambda_{1}\text{+}A/\lambda_{2}\\ \alpha_{2}=\sigma^{2}\;=\;\left(B-A^{2}\right)/\lambda_{2}^{2}\\ \alpha_{3}=\left(C-3AB+2A^{3}\right)/\left(\lambda_{2}^{3}\sigma^{3}\right)\\ \alpha_{4}=\left(D-4AC+6A^{2}B-3A^{4}\right)/\left(\lambda_{2}^{4}\sigma^{4}\right) \end{array}$$

where,

$$\begin{array}{l} A=1/\left(1+\lambda_{3}\right)-1/\left(1+\lambda_{4}\right)\\ B=1/\left(1+2\lambda_{3}\right)+1/\left(1+2\lambda_{4}\right)-2\beta \left(1+\lambda_{3},1+\lambda_{4}\right)\\ C=1/\left(1+3\lambda_{3}\right)-1/\left(1+3\lambda_{4}\right)-3\beta \left(1+2\lambda_{3},1+\lambda_{4}\right)\\ +\;3\beta \left(1+\lambda_{3},1+2\lambda_{4}\right)\\ D=1/\left(1+4\lambda_{3}\right)+1/\left(1+4\lambda_{4}\right)-4\beta \left(1+3\lambda_{3},1+\lambda_{4}\right)\\ +\;6\beta \left(1+2\lambda_{3},1+2\lambda_{4}\right)-4\beta \left(1+\lambda_{3},1+3\lambda_{4}\right) \end{array}$$

According to the mean, variance, skewness, and kurtosis of the expected generating distribution, the four parameters (λ_1_, λ_2_, λ_3_, λ_4_) of the corresponding GLD distribution can be calculated from the above equations, and then the random sample was generated from the distribution.

Next, the data set under the permanent missingness mechanisms was generated. The model for generating the missing data is defined as follows: Define the dummy variable *d*_*t*_ to describe whether the observation of target variable is missing at time *t* point, *d*_*t*_ = 0 means that the *y* is observed at time *t, d*_*t*_ = 1 represents *y* is missing at time *t*, and all missing after time *t*. The missing mechanism defined by the Probit regression model can be expressed as a formula:

$$\begin{array}{l} p_{t}\left(y_{1},y_{2},\ldots ,y_{t-1},y_{t}\right)=p\left(d_{k}=1|y_{1},y_{2},\ldots ,y_{t-1},y_{t}\right)\\ =\Phi \left(\beta_{1}y_{t-1}+\beta_{2}y_{t}-c\right) \end{array}$$

where *y*_*t*_ represents the target variable at time *t*, *p*_*t*_(*y*_1_, *y*_2_, ⋯ , *y*_*t*−1_, *y*_*t*_) is the conditional probability of missing at time *t, c* is the threshold of categorical variable *d*, and the specific value is calculated from the dropout rate. For non-random missingness mechanism, we set β_1_ = −0.5, β_2_ = 1; For random missingness mechanism, we set β_1_ = −0.5, β_2_ = 0 (Gad and Ahmed, 2007; Mazumdar et al., 2007; Soullier et al., 2010).

### Evaluation criteria

For each cell of the design, we simulated 500 sets of data. The performance of each method was evaluated according to five criteria, namely, (1) the convergence rates, (2) the bias and precision of the growth parameters estimates, (3) the bias and precision of the standard error, (4) the coverage probability (CP) of 95% confidence interval.

#### Bias

The bias was measured by the average difference between the estimation and the corresponding true value across replications. A negative bias value means an underestimation of parameter, while a positive bias value means an overestimation of parameter, the zero value means an unbiased estimation.

$$\begin{array}{l} \text{Bias }\left(\hat{\theta }\right)=\sqrt{\frac{1}{R}\overset{R}{\underset{r\;=\;1}{\sum }}{\left({\hat{\theta }}_{r}-\theta \right)}^{2}} \end{array}$$

#### Root mean square error (RMSE)

RMSE describes the difference between an estimated parameter and its true value. A lower RMSE means a smaller difference between the estimated value and the true value. RMSE is calculated as follows:

$$\begin{array}{l} \text{RMSE }\left(\hat{\theta }\right)=\sqrt{\frac{1}{R}\overset{R}{\underset{r\;=\;1}{\sum }}{\left({\hat{\theta }}_{r}-\theta \right)}^{2}} \end{array}$$

For the formula of the Bias and RMSE, θ is the true population parameter, ${\hat{\theta }}_{r}$ is the corresponding parameter estimate of the *r*th repeated, and *R* is the number of analyzed cases for each cell condition. Bias and RMSE were computed for both mean parameter estimates and estimated standard errors. For standard error estimate recovery, empirical standard deviations of each set of estimates were used as estimates of the true population values; these were computed separately for each design cell. Only successfully converged cases were used to estimate bias and RMSE for parameters and their standard errors.

#### The coverage probability (CP) of 95% confidence interval

This indicator shows the precision of an estimation and can reflect the parameter estimation precision and the corresponding estimated standard error to a certain extent. *CP* is calculated as follows:

$$\begin{array}{l} CP=\frac{1}{R}\overset{R}{\underset{r\;=\;1}{\sum }}CI_{95}\left({\hat{\theta }}_{r}\right) \end{array}$$

where $CI_{95}\left({\hat{\theta }}_{r}\right)=1$ if the value ${\hat{\theta }}_{r}$ falls within the 95% confidence interval; otherwise, $CI_{95}\left({\hat{\theta }}_{r}\right)=0$.

### Analytical methods

The study chose the MAR-based ML method and the MNAR-based Diggle–Kenward selection model method (MNAR-based DK method) to handle the missing data. For parameters estimation of the LGM, the ML estimation method was used for both missing data methods. For LGM, the parameters of interest were the means of latent variable (μ_*i*_ and μ_*s*_) and the variances of latent variable ($\sigma_{i}^{2}$ and $\sigma_{s}^{2}$). The μ_*i*_ and μ_*s*_ describe the average development trends of a sample, and the $\sigma_{i}^{2}$ and $\sigma_{s}^{2}$ indicate the development variations of individuals. In this study, the estimates of the parameters were obtained using Mplus7 (Muthén and Muthén, 2008–2012).

## Results

### Convergence

In all 64,000 replications, the non-convergence number for MAR-based ML method and MNAR-based DK method was only 2 and 19 times, respectively. This indicated that the difference of the convergence rate between two methods was negligible and the convergence was not a problem for both methods. The non-converged cases were removed prior to the analyses.

### Bias and precision of estimation

The bias and RMSE of parameters were used to evaluate the precision of estimators. The bias and RMSE of the four parameters of LGM including the mean of intercept (μ_*i*_), the mean of slope (μ_*s*_), the variance of intercept ($\sigma_{i}^{2}$), and the variance of slope ($\sigma_{s}^{2}$) are presented in Tables 1–**4**.

**Table 1:** **Bias and RMSE of means and standard errors of μ_***i***_ by different methods under different missingness mechanisms**.

	**Bias**				**RMSE**				**Bias of SE**				**RMSE of SE**			
	**MAR**		**MNAR**		**MAR**		**MNAR**		**MAR**		**MNAR**		**MAR**		**MNAR**	
**Factors**	**DK**	**ML**	**DK**	**ML**	**DK**	**ML**	**DK**	**ML**	**DK**	**ML**	**DK**	**ML**	**DK**	**ML**	**DK**	**ML**
**DISTRIBUTION**																
Normally distributed	0.002	0.001	−0.007	−0.047	0.055	0.054	0.054	0.074	−0.001	−0.001	0.000	0.000	0.003	0.003	0.003	0.003
Slightly skewed	0.001	0.001	−0.006	−0.046	0.054	0.053	0.054	0.073	0.000	0.000	0.000	0.000	0.003	0.003	0.004	0.003
Moderately skewed	−0.008	−0.001	−0.013	−0.050	0.054	0.052	0.055	0.075	0.001	0.001	0.000	0.000	0.005	0.005	0.004	0.004
Extremely skewed	−0.011	−0.002	−0.017	−0.052	0.055	0.053	0.056	0.077	0.001	0.001	−0.001	−0.001	0.007	0.006	0.005	0.005
**SAMPLE SIZE**																
100	−0.001	0.001	−0.012	−0.049	0.093	0.091	0.092	0.103	0.000	0.000	−0.001	0.000	0.010	0.010	0.010	0.009
300	−0.004	0.000	−0.010	−0.049	0.054	0.052	0.054	0.073	0.001	0.000	−0.001	0.000	0.004	0.004	0.003	0.003
500	−0.006	−0.002	−0.010	−0.049	0.042	0.040	0.042	0.065	0.001	0.001	0.000	0.000	0.002	0.002	0.002	0.002
1,000	−0.005	−0.001	−0.010	−0.049	0.030	0.029	0.031	0.058	0.000	0.000	0.000	0.000	0.001	0.001	0.001	0.001
**DROP RATIO (%)**																
5	−0.003	0.001	−0.008	−0.023	0.053	0.052	0.052	0.057	0.000	0.000	0.000	0.000	0.004	0.004	0.004	0.004
10	−0.006	−0.001	−0.011	−0.038	0.053	0.052	0.054	0.066	0.001	0.001	−0.001	−0.001	0.005	0.004	0.004	0.004
20	−0.005	0.000	−0.013	−0.061	0.056	0.053	0.056	0.082	0.000	0.000	0.000	0.000	0.004	0.004	0.004	0.004
40	−0.002	0.000	−0.010	−0.074	0.057	0.055	0.058	0.095	0.001	0.000	0.000	0.000	0.004	0.004	0.005	0.004
All Replications	−0.004	0.000	−0.010	−0.049	0.055	0.053	0.055	0.075	0.000	0.000	0.000	0.000	0.004	0.004	0.004	0.004

Under the MAR mechanism, the precision of parameter estimation conducted using the MAR-based ML method was slightly better than that of the MNAR-based DK method; while under the MNAR mechanism, the MNAR-based DK method performed better than the MAR-based ML method. This is because the MAR mechanism better fits the assumptions of the ML method on missingness mechanism, while the MNAR mechanism better fits the assumptions of the Diggle–Kenward selection model on missingness mechanism. The precision of parameter estimation of both methods can be improved with the increasing of the sample size.

As shown in Table 1, under the MAR mechanism, the differences of bias and RMSE for the mean of intercept between the MNAR-based DK method and the MAR-based ML method were small, and the estimation precision of the intercept mean was affected by neither the dropout rate nor the non-normal degree of the target variable. However, under the MNAR mechanism, the differences of bias and RMSE between two methods were relatively large, which increased with the increasing of the dropout rate, but the differences were unaffected by the non-normal degree of the target variable. Overall, for the mean of intercept, the MNAR-based DK method can obtain robust estimation even under the MAR mechanism. However, under the MNAR mechanism, the ML method seriously underestimated the intercept mean, except for the case with a dropout rate of <5%.

The results for the mean of slope was similar to the intercept mean (see Table 2). Under the MAR missingness mechanism, the bias and RMSE of MAR-based ML method were both lower than those of the MNAR-based DK method, and the MNAR-based DK method slightly underestimated the slope mean. Under the MNAR missingness mechanism, the bias and RMSE of the MAR-based ML method were higher than those of the MNAR-based DK Method, and MAR-based ML method seriously underestimated the mean of the slope. The MAR-based ML method was more sensitive to the choice of the missingness mechanisms. The bias and RMSE of MAR-based ML method were not affected by the degree of non-normality, but the bias and RMSE of the MNAR-based DK method increased with the increasing of non-normality degree. The dropout rate was the main influential factor of the slope mean estimators, and the estimation precision of both methods dramatically decreased when the dropout rate reached above 20%. Moreover, the differences between the two methods increased with the increasing of the dropout rate.

**Table 2:** **Bias and RMSE of means and standard errors of μ_***s***_ by different methods under different missingness mechanisms**.

	**Bias**				**RMSE**				**Bias of SE**				**RMSE of SE**			
	**MAR**		**MNAR**		**MAR**		**MNAR**		**MAR**		**MNAR**		**MAR**		**MNAR**	
**Factors**	**DK**	**ML**	**DK**	**ML**	**DK**	**ML**	**DK**	**ML**	**DK**	**ML**	**DK**	**ML**	**DK**	**ML**	**DK**	**ML**
**DISTRIBUTION**																
Normally distributed	0.002	0.000	−0.006	−0.077	0.035	0.021	0.033	0.081	−0.009	−0.001	−0.006	0.000	0.013	0.002	0.011	0.002
Slightly skewed	−0.005	−0.004	−0.013	−0.078	0.037	0.021	0.034	0.081	−0.009	−0.001	−0.005	0.000	0.012	0.002	0.013	0.002
Moderately skewed	−0.027	−0.009	−0.027	−0.077	0.053	0.023	0.043	0.080	−0.015	0.000	−0.007	0.000	0.013	0.002	0.006	0.002
Extremely skewed	−0.037	−0.011	−0.029	−0.076	0.065	0.024	0.044	0.080	−0.020	−0.001	−0.007	−0.001	0.029	0.002	0.011	0.002
**SAMPLE SIZE**																
100	−0.022	−0.006	−0.018	−0.077	0.082	0.037	0.060	0.085	−0.027	−0.002	−0.012	−0.001	0.038	0.005	0.024	0.004
300	−0.017	−0.006	−0.019	−0.077	0.046	0.022	0.036	0.080	−0.013	0.000	−0.006	0.000	0.016	0.002	0.008	0.002
500	−0.015	−0.006	−0.020	−0.077	0.035	0.017	0.031	0.079	−0.008	0.000	−0.005	0.000	0.010	0.001	0.005	0.001
1,000	−0.014	−0.006	−0.020	−0.077	0.027	0.013	0.026	0.078	−0.005	0.000	−0.003	0.000	0.005	0.001	0.003	0.001
**DROP RATIO (%)**																
5	−0.006	−0.001	−0.009	−0.023	0.020	0.015	0.019	0.028	−0.001	0.000	−0.001	0.000	0.002	0.001	0.001	0.001
10	−0.010	−0.003	−0.014	−0.042	0.027	0.016	0.025	0.045	−0.003	0.000	−0.002	0.000	0.005	0.001	0.003	0.001
20	−0.016	−0.005	−0.021	−0.077	0.042	0.021	0.036	0.080	−0.008	−0.001	−0.004	0.000	0.012	0.002	0.006	0.002
40	−0.035	−0.014	−0.032	−0.167	0.102	0.037	0.073	0.170	−0.039	−0.002	−0.019	−0.001	0.050	0.004	0.032	0.004
All Replications	−0.017	−0.006	−0.019	−0.077	0.048	0.022	0.038	0.080	−0.013	−0.001	−0.006	0.000	0.017	0.002	0.010	0.002

As can be seen from Table 3, for the variance of intercept, there was a slight difference between the two methods under either the MAR or the MNAR mechanism. The non-normal degree of the target variable had a significant impact on the estimation precision of the intercept variance: the precision of the parameter estimation was similar to that under the normal distribution when data departed slightly from the normal distribution. However, the parameter estimation precision declined as data departed further from the normal distribution.

**Table 3:** **Bias and RMSE of means and standard errors of $\sigma_{\text{i}}^{2}$ by different methods under different missingness mechanisms**.

	**Bias**				**RMSE**				**Bias of SE**				**RMSE of SE**			
	**MAR**		**MNAR**		**MAR**		**MNAR**		**MAR**		**MNAR**		**MAR**		**MNAR**	
**Factors**	**DK**	**ML**	**DK**	**ML**	**DK**	**ML**	**DK**	**ML**	**DK**	**ML**	**DK**	**ML**	**DK**	**ML**	**DK**	**ML**
**DISTRIBUTION**																
Normally distributed	−0.004	−0.002	−0.003	0.014	0.078	0.078	0.074	0.077	−0.001	−0.001	−0.001	−0.001	0.009	0.008	0.008	0.008
Slightly skewed	0.009	0.008	−0.006	0.007	0.079	0.078	0.076	0.077	−0.002	−0.001	−0.002	−0.001	0.009	0.008	0.012	0.008
Moderately skewed	0.036	0.026	−0.044	−0.029	0.132	0.127	0.108	0.109	−0.049	−0.048	−0.021	−0.028	0.048	0.049	0.026	0.031
Extremely skewed	0.037	0.023	−0.076	−0.054	0.178	0.172	0.129	0.132	−0.097	−0.095	−0.029	−0.042	0.099	0.096	0.032	0.046
**SAMPLE SIZE**																
100	0.017	0.009	−0.034	−0.018	0.194	0.192	0.155	0.166	−0.063	−0.062	−0.021	−0.034	0.073	0.071	0.041	0.047
300	0.019	0.014	−0.033	−0.016	0.114	0.110	0.095	0.096	−0.035	−0.034	−0.013	−0.016	0.039	0.037	0.017	0.020
500	0.019	0.014	−0.032	−0.015	0.089	0.085	0.078	0.077	−0.027	−0.025	−0.011	−0.013	0.029	0.028	0.013	0.015
1,000	0.022	0.017	−0.031	−0.014	0.071	0.067	0.060	0.058	−0.024	−0.023	−0.007	−0.009	0.025	0.024	0.008	0.010
**DROP RATIO (%)**																
5	0.009	0.003	−0.024	−0.018	0.110	0.107	0.088	0.090	−0.038	−0.037	−0.016	−0.019	0.042	0.041	0.020	0.023
10	0.014	0.008	−0.031	−0.021	0.113	0.108	0.092	0.094	−0.038	−0.036	−0.014	−0.019	0.043	0.041	0.018	0.023
20	0.020	0.016	−0.035	−0.017	0.113	0.109	0.098	0.098	−0.033	−0.032	−0.013	−0.017	0.037	0.035	0.018	0.022
40	0.033	0.028	−0.040	−0.007	0.132	0.130	0.110	0.114	−0.039	−0.039	−0.008	−0.018	0.042	0.044	0.022	0.024
All Replications	0.019	0.013	−0.032	−0.016	0.117	0.114	0.097	0.099	−0.037	−0.036	−0.013	−0.018	0.041	0.040	0.019	0.023

According to the results shown in Table 4, under the MNAR mechanism, there was no difference in the bias and RMSE between the MNAR-based DK method and the MAR-based ML method; The RMSE of the slope variance estimators increased as the dropout rate increased, but it was not affected by the non-normal degree of the target variable. Under the MAR mechanism, the differences in Bias and RMSE between the MNAR-based DK method and the MAR-based ML method increased with the increasing of both the dropout rate and the non-normal degree of the target variable. Overall, the results indicate that the MAR-based ML method can obtain robust estimation even under the MNAR mechanism, while the MNAR-based DK method can only produce robust estimations under the MAR mechanism when the dropout rate was below 20% or the target variable departed only slightly from normal distribution.

**Table 4:** **Bias and RMSE of means and standard errors of $\sigma_{\text{s}}^{2}$ by different methods under different missingness mechanisms**.

	**Bias**				**RMSE**				**Bias of SE**				**RMSE of SE**			
	**MAR**		**MNAR**		**MAR**		**MNAR**		**MAR**		**MNAR**		**MAR**		**MNAR**	
**Factors**	**DK**	**ML**	**DK**	**ML**	**DK**	**ML**	**DK**	**ML**	**DK**	**ML**	**DK**	**ML**	**DK**	**ML**	**DK**	**ML**
**DISTRIBUTION**																
Normally distributed	0.001	0.000	0.000	0.001	0.009	0.008	0.009	0.008	−0.001	0.000	0.000	0.000	0.003	0.001	0.003	0.001
Slightly skewed	0.001	−0.001	0.000	0.002	0.010	0.008	0.009	0.009	−0.001	0.000	0.000	0.000	0.003	0.001	0.004	0.001
Moderately skewed	0.003	0.000	0.003	0.003	0.012	0.008	0.010	0.010	−0.002	0.000	−0.001	−0.001	0.003	0.001	0.002	0.002
Extremely skewed	0.005	0.000	0.003	0.004	0.015	0.008	0.010	0.011	−0.004	0.000	−0.001	−0.001	0.008	0.001	0.003	0.002
**SAMPLE SIZE**																
100	0.005	−0.001	0.003	0.003	0.022	0.014	0.016	0.016	−0.005	−0.001	−0.001	−0.001	0.012	0.003	0.008	0.004
300	0.002	0.000	0.001	0.002	0.011	0.008	0.009	0.009	−0.002	0.000	0.000	0.000	0.003	0.001	0.002	0.001
500	0.001	0.000	0.001	0.002	0.008	0.006	0.007	0.007	−0.001	0.000	0.000	0.000	0.002	0.001	0.001	0.001
1,000	0.001	0.000	0.001	0.003	0.005	0.004	0.005	0.005	0.000	0.000	0.000	0.000	0.001	0.000	0.000	0.000
**DROP RATIO (%)**																
5	0.001	0.000	0.000	0.000	0.006	0.006	0.006	0.006	0.000	0.000	0.000	0.000	0.001	0.001	0.001	0.001
10	0.001	0.000	0.001	0.001	0.007	0.006	0.007	0.007	0.000	0.000	0.000	0.000	0.001	0.001	0.001	0.001
20	0.002	0.000	0.001	0.002	0.009	0.008	0.008	0.008	−0.001	0.000	0.000	0.000	0.003	0.001	0.002	0.001
40	0.006	0.000	0.005	0.007	0.023	0.013	0.015	0.017	−0.007	−0.001	−0.001	−0.002	0.013	0.003	0.008	0.004
All Replications	0.002	0.000	0.002	0.002	0.011	0.008	0.009	0.009	−0.002	0.000	0.000	0.000	0.004	0.001	0.003	0.002

### Estimated standard error

The bias and RMSE of standard error were used to evaluate the efficiency of the estimates, and the results are shown in Tables 1–4. For all parameters, the bias and RMSE of SE decreased as the sample size increased.

For the SE of the intercept mean (see Table 1), the bias and RMSE were very small, and the differences between the MAR-based ML method and the MNAR-based DK method were negligible in all conditions. The dropout rate and the non-normal degree of the target variable had no effect on the estimation efficiency of the intercept mean.

For the SE of the slope mean (see Table 2), the bias and RMSE of the MAR-based ML method were smaller than those of the MNAR-based DK method. The bias and RMSE were not affected by the non-normal degree of the target variable for the MAR-based ML method. In contrast, the non-normal degree of the target variable affected the bias and RMSE for the MNAR-based DK method, especially under MAR missingness mechanisms. The bias and RMSE increased as the dropout rate increased. However, it can be seen from Table 2 that the standard error was underestimated, especially for the MNAR-based DK method under the MAR missingness mechanism.

For the SE of the intercept variance and the slope variance, the bias and RMSE were very small, and the differences between MAR-based ML method and the MNAR-based DK method were also small. Moreover, both methods underestimated the standard error of the intercept variance under whichever missingness mechanism.

### The 95% coverage probability (95% CP)

The study used the normal distribution method to construct the 95% confidence intervals for the estimated means and variances of the growth parameters. Table 5 provides the 95% coverage probabilities results.

**Table 5:** **The 95% coverage probability for parameters of latent growth model (%)**.

	**MAR**								**MNAR**							
	***μ_**i**_***		***μ_**s**_***		***σ_**i**_***^**2**^		***σ_**s**_***^**2**^		***μ_**i**_***		***μ_**s**_***		***σ_**i**_***^**2**^		***σ_**s**_***^**2**^	
**Factors**	**DK**	**ML**	**DK**	**ML**	**DK**	**ML**	**DK**	**ML**	**DK**	**ML**	**DK**	**ML**	**DK**	**ML**	**DK**	**ML**
**DISTRIBUTION**																
Normally distributed	93.7	94.9	83.8	94.6	92.1	94.5	91.2	94.5	90.8	80.9	90.1	**39.2**	94.2	94.4	92.7	94.5
Slightly skewed	94.3	95.3	86.9	94.5	92.7	94.3	90.9	94.0	88.9	**79.2**	90.4	**38.8**	94.6	94.7	92.6	94.5
Moderately skewed	95.1	95.2	90.0	93.7	80.0	81.8	90.0	94.5	86.0	**77.8**	83.9	**37.6**	**78.9**	81.2	91.4	94.2
Extremely skewed	94.8	94.6	89.0	92.7	70.8	72.5	89.3	94.4	84.6	**77.2**	80.0	**38.8**	**65.9**	**71.6**	91.0	94.3
**SAMPLE SIZE**																
100	94.6	94.7	89.8	94.0	84.4	86.3	84.3	93.5	92.2	90.2	89.2	**70.3**	85.8	86.3	87.5	93.9
300	94.7	95.1	89.2	94.0	84.5	86.5	91.9	94.6	90.2	83.7	88.5	**41.4**	85.1	86.2	93.1	94.6
500	94.4	94.9	88.0	94.0	84.1	85.7	92.9	94.5	87.5	**77.3**	86.5	**28.2**	84.1	86.3	93.7	94.9
1,000	94.2	95.4	83.2	93.4	82.6	84.6	92.4	94.7	80.4	**63.9**	80.2	**14.5**	**78.6**	83.2	93.3	94.2
**DROP RATIO (%)**																
5	95.0	95.1	89.2	94.8	83.0	85.0	92.2	94.4	90.6	91.3	88.5	**72.4**	83.9	85.6	93.1	94.6
10	94.7	94.9	87.6	94.7	83.0	85.6	91.4	94.2	88.2	87.4	85.2	**45.9**	84.2	86.0	93.2	94.7
20	94.7	95.5	87.1	93.3	84.0	86.1	90.5	94.5	85.3	**75.1**	85.4	**22.8**	83.4	85.8	92.2	94.5
40	93.5	94.7	86.3	92.7	85.7	86.3	87.4	94.2	86.3	**61.3**	85.3	**13.3**	82.1	84.5	89.3	93.9
All Replications	94.5	95.0	87.5	93.9	83.9	85.8	90.4	94.3	87.6	**78.8**	86.1	**38.6**	83.4	85.5	91.9	94.4

*The value of 95% coverage probability that under 80% is printed in bold*.

Table 5 shows that under the MAR mechanism, the 95% CP for the intercept mean obtained using the MNAR-based DK method and the MAR-based ML method stood at 94.5 and 95.0%, respectively, with the difference being very small. Under the MNAR mechanism, the 95% CP of the two methods were 87.6 and 78.7%, respectively, and the CP of the MAR-based ML method was lower than that of the MNAR-based DK method. Under the MNAR mechanism, the CP of MAR-based ML method decreased with increasing degree of non-normality, sample size, and the dropout rate.

For the CP of the slope mean, under the MAR mechanism, the 95% CP obtained using the MNAR-based DK method and the MAR-based ML method were 87.5 and 93.9%, respectively. Under the MNAR mechanism, the 95% CP for the two methods were 86.1 and 38.6%, respectively, which were both under the desired level of 95%. The MNAR-based DK method yielded much higher coverage rates than the MAR-based ML method, the coverage rates of which were far below 95%.

For the estimation of the intercept variance under the MAR mechanism, the 95% CP obtained using the MNAR-based DK method and the MAR-based ML method were 83.9 and 85.8%, respectively, representing a very small difference. Under the MNAR mechanism, the coverage probability for the two methods were 83.4 and 85.5%, respectively, which had little difference but were both under the desired level of 95%.

For the estimation of the slope variance under the MAR mechanism, the 95% CP calculated using the MNAR-based DK method and the MAR-based ML method were 90.4 and 94.3%, respectively. Under the MNAR mechanism, the 95% CP for the two methods were 91.9 and 94.4%, respectively. Therefore, the difference between the slope variances of the two methods was small.

## Discussion and suggestions

### Discussion

The methods used for handling missing data in longitudinal studies are normally based on certain missingness mechanism assumptions. Appropriate methods should be chosen to accommodate different mechanisms. The untestable assumptions of the MNAR mechanism have posed some difficulties for the selection of methods, and thus this issue is hotly debated in the research field. This study focused on the following three questions: (1) Under the MAR mechanism, can the MNAR-based Diggle–Kenward selection model yield similar results to the ML method? (2) Under the MNAR mechanism, can the MAR-based ML method yield similar results to the Diggle–Kenward selection model? (3) Will the factors, such as dropout rate, normal distribution assumption, and sample size affect the estimation results?

The MAR-based ML method delivers better results than the MNAR-based DK method under the MAR mechanism; while the MNAR-based DK method performs better than the MAR-based ML method under the MNAR mechanism. This result further demonstrates the importance of choosing the right model for dealing with missing data. However, the missing data mechanism is usually not known in practice, and no reliable method exists to determine whether the missingness mechanism assumptions are established. A study on the robustness of different methods under different missingness mechanism assumptions can provide valuable insights into the method selection in real-case studies.

We concluded in the study, under the MAR mechanism, there was a slight difference between the results obtained by the two methods for the three parameters of LGM including mean of intercept, variance of intercept, and variance of slope, especially in the case of large sample size. However, for the mean of slope, the MNAR-based DK method underestimated the mean and the standard error, and the difference between MAR-based ML method and the MNAR-based DK method was obvious, but the difference reduced with the increasing of the sample size. This finding indicates that for MAR data, the MNAR-based DK method can provide a robustness result in the estimation of slope mean under large sample sizes. However, we should notice that the coverage rate of the MNAR-based DK method was lower than expected rate of 95% due to the underestimated SE.

Under the MNAR mechanism, there was a slight difference in the intercept variance and slope variance obtained by the two methods, respectively. This suggests that even when the data are MNAR, the use of the MAR-based ML method will not create extremely biased estimates for the variance of growth parameters. However, there were remarkable differences between the two methods in the estimation of intercept mean and slope mean, the MAR-based ML method produced highly biased estimates even under large sample size. This shows that even under large sample size, the MAR-based ML method has a large bias in the estimation of intercept mean and slope mean under the MNAR missingness mechanism, and a similar conclusion can be drawn for the 95% coverage probability of confidence interval, especially for estimation of the slope mean.

It should be noted that when the dropout rate is small (for example under 10%), there was a slight difference between the two methods. Generally, the MNAR-based DK method is less influenced by missingness mechanisms. But when choosing analytical methods in practice, factors, such as the sample size, the skewness degree of the target variable, and the dropout rate, should be taken into consideration simultaneously. Lu et al. (2013) and Lu and Zhang (2014) concluded that the wrong definition of the missingness mechanism may lead to the wrong conclusion based on the latent growth model and the mixed growth model, respectively, which is consistent with the result obtained (Lu et al., 2013; Lu and Zhang, 2014).

The performance of each model was also examined in making estimations when the assumptions on the normal distribution of the target variable are violated. For the bias and RMSE of the intercept mean, the non-normal degree had no impact on the two methods. For the slope mean, the bias and RMSE of MAR-based ML method was not affected by the degree of non-normality, and the conclusions agree with previous studies (Enders, 2001; Tacksoo et al., 2009; Yuan et al., 2012). But the MNAR-based DK method was more greatly impacted by the non-normal degree. For the intercept variance and SE, both methods were affected greatly by the non-normal degree. For the slope variance and SE, both methods were hardly influenced by the non-normal degree. Overall, the MNAR-based DK method is more likely to be influenced by the distribution of non-normal degree of the target variable. As Muthén et al. (2011), and Enders (2011b) pointed out that the parameter estimation was more dependent on the assumption of normality for MNAR missingness data because the missingness mechanism depended on the distribution of the variables.

Finally, it is worth noting that the dropout rate is an important factor that affects the accuracy and effectiveness of parameter estimation. In the longitudinal study, the intercept-related parameters often define the initial state of the target variable, therefore it should not be affected by the later target variables. The slope parameters are used to describe the developmental changes, the dropout rate and the way how to deal with the missingness have a great impact on it. The results of our study also supported these conclusions. It is found that for the slope mean, slope variance and their corresponding standard errors, the estimated bias of the parameter increases with the increase of the dropout rate. The difference between the two different mechanisms can be ignored when the dropout rate is <10%. However, a model based on the un-proper missingness mechanism would result in a larger estimated bias if the dropout rate exceeds 10%.

### Suggestions and further research

As the theoretical assumptions of the MNAR mechanism are more stringent compared with MCAR and MAR, a higher level of care should be taken when deciding the methods for dealing with MNAR data. For research data with missing values, it is essential to fully understand the potential causes for the missing data and perform a comprehensive analysis on the data by following a specific procedure. Based on the findings of the simulation study, we recommend that considerations should be taken of the potential influences of the non-normal degree of the target variable, the dropout rate, and the sample size on parameter estimations when conducting analysis.

First, verify if the target variable follows a normal distribution. If the target variable is moderately or highly skewed from normal distribution, a large sample size may be needed or we should do further research to use other robustness estimate method to handle the non-normal missing data.

Second, check the level of dropout rate of longitudinal data. If the dropout rate is below 10%, the MAR-based ML method can be used based on the “simplification of model” principle.

Third, if it cannot be decided whether missing data are MCAR, and/or if it can be judged from existing studies or experience that MNAR data might be present at a high level, and then it is necessary to perform sensitivity analysis (Graham et al., 1997; Carpenter et al., 2007; Jamshidian and Yuan, 2012; Morenobetancur and Chavance, 2016). Many methodologists recommend that different models or methods should be used to analyze data that may contain MNAR-values in order to examine the differences between the results yielded by these different methods (Enders, 2011a; Muthén et al., 2011).

Forth, compare the results derived using the different methods and choose the most reasonable assumption to interpret the results of data analysis. For ML method and DK method, if the conclusions are consistent, the results under the MAR mechanism are also considered to be reliable. If the conclusions under MNAR assumptions are inconsistent with those under MAR assumptions, then the results under the MNAR mechanism are considered to be more credible especially for large sample size. The reason is that the MAR hypothesis can often be seen as a special case of the MNAR. For example, in the DK model, if it is assumed that the effect of the target variable at time t on the missingness dummy variable is zero, the ML method assumption is met.

### Limitation

This study mainly discussed the comparison of the ML method under the assumption of MAR deletion mechanism and the DK selection model under the hypothesis of the MNAR deletion mechanism. However, it should be noted that even if the analysis based on MNAR mechanism, there are many other different models based on different assumptions such as selection models and pattern-mixture models. Subsequent studies would focus on more other MNAR-based models and compare their performances under the MNAR mechanism. In addition, for the estimation method, we should consider more methods, such as two-stage ML method (Yuan and Lu, 2008) and Bayesian method (Lu et al., 2013), among others. For the model selection criteria, the model fitness indexes such as AIC, BIC, and DIC, should be considered for latent growth models with missing data.

## Author contributions

ML was mainly responsible for the analysis and the interpretation of data and drafting the research. NC was mainly responsible for the analysis and the interpretation of data and drafting the research. YC helped a lot providing useful suggestions on revising the article. HL was mainly responsible for the design of the research, the analysis, and the interpretation of data and drafting the research.

### Conflict of interest statement

The authors declare that the research was conducted in the absence of any commercial or financial relationships that could be construed as a potential conflict of interest.
